# Combining lung ultrasound and Wells score for diagnosing pulmonary embolism in critically ill COVID-19 patients

**DOI:** 10.1007/s11239-020-02323-0

**Published:** 2020-11-03

**Authors:** Viviane Zotzmann, Corinna N. Lang, Tobias Wengenmayer, Xavier Bemtgen, Bonaventura Schmid, Katharina Mueller-Peltzer, Alexander Supady, Christoph Bode, Daniel Duerschmied, Dawid L. Staudacher

**Affiliations:** 1 Department of Cardiology and Angiology I, Heart Center Freiburg University, Faculty of Medicine, University of Freiburg, Hugstetter Str. 55, 79106 Freiburg, Germany; 2 Department of Medicine III (Interdisciplinary Medical Intensive Care), Medical Center, Faculty of Medicine, University of Freiburg, Freiburg, Germany; 3 Department of Diagnostic and Interventional Radiology, Faculty of Medicine, University of Freiburg, Freiburg, Germany; 4 Department of Emergency Medicine, Medical Center, Faculty of Medicine, University of Freiburg, Freiburg, Germany; 5 Heidelberg Institute of Global Health, University of Heidelberg, Heidelberg, Germany

**Keywords:** COVID-19, Lung ultrasound, CTPA, Pulmonary embolism, SARS-CoV2, Wells score

## Abstract

Subpleural consolidations have been found in lung ultrasound in patients with COVID-19, possibly deriving from pulmonary embolism (PE). The diagnostic utility of impact of lung ultrasound in critical-ill patients with COVID-19 for PE diagnostics however is unclear. We retrospectively evaluated all SARS-CoV2-associated ARDS patients admitted to our ICU between March 8th and May 31th 2020. They were enrolled in this study, when a lung ultrasound and a computed tomography pulmonary angiography (CTPA) were documented. In addition, wells score was calculated to estimate the probability of PE. The CTPA was used as the gold standard for the detection of PE. Twenty out of 25 patients met the inclusion criteria. In 12/20 patients (60%) (sub-) segmental PE were detected by CT-angiography. Lung ultrasound found subpleural consolidations in 90% of patients. PE-typical large supleural consolidations with a size ≥ 1 cm were detectable in 65% of patients and were significant more frequent in patients with PE compared to those without (p = 0.035). Large consolidations predicted PE with a sensitivity of 77% and a specificity of 71%. The Wells score was significantly higher in patients with PE compared to those without (2.7 ± 0.8 and 1.7 ± 0.5, respectively, p = 0.042) and predicted PE with an AUC of 0.81. When combining the two modalities, comparing patients with considered/probable PE using LUS plus a Wells score ≥ 2 to patients with possible/unlikely PE in LUS plus a Wells score < 2, PE could be predicted with a sensitivity of 100% and a specificity of 80%. Large consolidations detected in lung ultrasound were found frequently in COVID-19 ARDS patients with pulmonary embolism. In combination with a Wells score > 2, this might indicate a high-risk for PE in COVID-19.

**Electronic supplementary material:**

The online version of this article (10.1007/s11239-020-02323-0) contains supplementary material, which is available to authorized users.

## Highlights

- Large subpleural consolidations ≥ 1cm detected in lung ultrasound were found frequently in COVID-19 ARDS patients with pulmonary embolism. In combination with a Wells score > 2, PE could be predicted in COVID-19 ARDS with a sensitivity of 100% and a specificity of 80%.

## Introduction

In hospitalized patients infected with the severe acute respiratory syndrome coronavirus 2 (SARS-CoV-2), respiratory failure is a common complication. Several case reports and retrospective registry analyses report a high rate of thrombotic complications including pulmonary embolism (PE) and microvascular thrombosis [1–3]. In a recent analysis, patients with severe coronavirus disease 2019 (COVID-19) who were treated with low-molecular weight heparin (LMWH) had a lower 28-day mortality compared with similarly-ill patients that were not treated with LMWH [4].

Since the beginning of the outbreak multiple studies evaluating imaging techniques described bilateral ground-glass opacities, crazy-paving and air-space consolidations in peripheral and basal distribution in patients with COVID-19 pneumonia [5, 6]. Zieleskiewicz et al. were able to show that the assessment of the LUS score was an indication of the severity of pneumonia, which was evaluated by a chest CT scan [7].

Peng et al. and Volpicelli et al. described COVID-19-typical signs in lung ultrasound (LUS). These signs can already be useful in the emergency department for risk stratification and for underpinning the diagnostic assurance [8–10].

Subpleural consolidations are among those COVID-19-specific sonographic signs, described by Peng et.al [8]. As elegantly described earlier by Reissig et al., these consolidations are well known and described sonographic criteria for peripheral or segmental PE with high sensitivity and specificity (76.9% and 91.3%, respectively). Therefore, it might be possible that these COVID-19-typical ultrasound-signs not only mimic but rather actually picture a peripheral or (sub-)segmental PE [11].

In order to address this issue of immediate therapeutic relevance, we conducted a retrospective registry analyses to investigate if lung ultrasound in critical-ill COVID-19-patients could help to diagnose PE manifestations.

## Methods

This is an investigator-initiated retrospective non-interventional registry study. All data were collected retrospectively from patient records at the University of Freiburg Medical Center.

### Study population and data collection

We recruited all patients reverse transcriptase polymerase chain reaction (rtPCR)-confirmed SARS-CoV-2 infection treated at the medical intensive care unit (ICU) at the University of Freiburg Medical Center between March 8th and May 31th, 2020 if they fulfilled all of the following inclusion criteria:

1. respiratory failure as defined by ARDS with a Horowitz index less than 100 mmHg due to SARS-CoV-2
2. lung ultrasound and
3. contrast enhanced CT-scan with pulmonary angiography (CTPA) were performed and documented.

All data for this study was taken from the electronic patients files. As data were collected retrospectively, no interventions were applied for the purpose of this study and all patients were treated according to current treatment standards and guidelines.

### Diagnostic pathway during ICU course

Our ICU is located at a university hospital offering a 24/7 ECMO center specialized in acute respiratory distress syndrome (ARDS). ARDS treatment is performed according to current guidelines, including early mobilization or prone positioning and early spontaneous breathing in patients without desynchronization with the ventilator [12]. In case of severe pulmonary failure, a multidisciplinary team including at least the intensivist in charge, an ECMO specialist, a registered nurse and a perfusionist decide about extracorporeal membrane oxygenation (ECMO) in severe courses where invasive mechanical ventilation is not sufficient. During the SARS-CoV2 pandemic, daily LUS was encouraged by local standard operating procedures when deterioration in respiratory function was evident.Imaging (sonography, including LUS, echocardiography and sonography) was performed by experienced intensivists. A CTPA was performed when indicated by the intensivist and radiologist in charge.

### Lung ultrasound (LUS)

Experienced intensivists with advanced knowledge in sonography carried out the LUS. Evaluation and assessment of the lung sonography findings was mostly done with knowledge of the d-dimer value, but before performing a CTPA and therefore prospectively blinded with regard to the CTPA result. LUS examination was carried out using a Philips CX50 echocardiography machine with a multifrequency probe C5-1 (5-1 MHz) or L12-3 (12-3 MHz). Alternatively, a Philips Sparq with a multifrequency probe C6-2 (6-2 MHz) or L12-4 (12-4 MHz) was used. Due to our local standard, we used an adjusted BLUE protocol for investigating the lungs. The BLUE protocol [13] is a standardized diagram for the rapid identification of 97% of the causes of dyspnea in adult patients (pulmonary edema, pneumonia, PE, COPD, asthma, pneumothorax). Ultrasound examinations were performed along the midclavicular line in the bilateral anterior chest wall and the scapular line and interscapular regions in the posterior chest wall—each right and left side of the chest—at the bedside. Since the mechanically ventilated intensive care patients can usually only be examined either lying on the front or back, examination of all 12 lung fields was only possible in individual cases, mostly only 8 fields could be examined reliably. Furthermore, we focused on the COVID-19-typical signs as described before [8], such as multiple B-lines (comet-tail artefacts) in a variety of patterns (focal, multifocal and confluent), a thickening of the pleural line with irregularity and consolidations in a variety of patterns (see Fig. 2). On a LUS survey sheet we documented the number of fields examined as well as the patterns described above (B-Lines, consolidations, pleura-irregularities) for each individual field. Finally, the summary of the examination assessed whether it was suitable for COVID-19 and whether PE was highly likely, probable, possible or unlikely.

The diagnosis of PE suggested by Mathis et al., based on the number and size of the subpleural consolidations, was used but slightly modified. Mathis et al. describe consolidations with a size of more than 5 mm as typical for PE [14]. Due to the pronounced pleural changes in COVID-19 with a significant thickening of the pleura, we only considered triangular consolidations ≥ 1 cm as PE-typical, < 1 cm as non-typical for PE. The following criteria for detection of PE diagnosis were used.

PE is considered highly likely when two or more characteristic triangular lesions (≥ 1 cm) were demonstrated; PE is considered probable: if one characteristic triangular lesion (≥ 1 cm) was detected; PE is considered possible: if two (or more) non-typical lesions (< 1 cm) were detected; PE is considered unlikely: neither typical nor atypical consolidations.

To assess hemodynamic relevance of the PE, additionally, right heart echocardiography and sonography of the vena cava were performed.

### CTPA examination protocol and imaging analysis

Our local standard operating procedure strongly recommended that all patients with ARDS and SARS-CoV2 infection should undergo CTPA, unless endangering patient heath. CTPA scans were performed using a commercial CT scanner (SOMATOM Definition Flash; Siemens Healthineers GmbH, Forchheim, Germany) with the following scanning parameters: tube voltage, 100 kV; tube current, 90 mAs; rotation time, 0.28 s. 128 × 0.6 mm collimation with automated dose modulation (CARE dose4D, Siemens Healthineers GmbH, Forchheim, Germany). Patients without extracorporeal membrane oxygenation (ECMO) received the standard pulmonary angiography protocol with bolus-tracking method of 70 ml contrast agent (Imeron 400, Bracco Imaging, Germany). To consider an altered blood flow in patients with ECMO device the amount of contrast agent was adjusted to 100 ml and the ROI was placed in the air. The scan was manually started when an adequate contrast was visually detected in the pulmonary trunk. If tolerated by the patient ECMO flow was reduced to 70 to 50% of the initial value after scout acquisition for the time of the contrast-enhanced scan. Each pulmonary lung segment was separately evaluated for parenchymal abnormalities (ground-glass opacities and/or crazy-paving pattern, and air space consolidation) and PE.

A segmental or subsegmental PE was defined as central filling defect within a vessel surrounded by contrast material when orthogonal or parallel to the long axis of the vessel as well as eccentric filling defect rendering an acute angle with the vessel wall as well as complete occlusion of a dilated vessel [15].

### Wells score

The Wells score is a well-established screening tool for PE and is used in everyday care to assess the clinical pre-Test probability at our institution [16]. The following questions are scored: Clinical signs and symptoms of deep vein thrombosis (DVT)? (+ 3), PE is #1 diagnosis or equally likely? (+ 3), heart rate > 100 bpm? (+ 1.5), Immobilization at least 3 days OR surgery in the previous 4 weeks? (+ 1.5), previous objectively diagnosed PE or DVT? (+ 1.5), hemoptysis? (+ 1) and malignancy with treatment within 6 months or palliative? (+ 1). The largest study [16] using a three-tier Model and demonstrated risk stratification with: (a) Low score of 1–2 points having a 1.3% prevalence, (b) Moderate score of 2–6 points having a 16.2% prevalence or (c) High score of > 6 points having a 37.5% prevalence. In order to reduce inter-observer variance for our research, the Wells score was retrospectively re-assessed for the day of CTPA by a single intensivist considering all available data.

### Statistical analysis and ethics

Statistical analyses were performed using SPSS, version 23.0, (IBM, New York City, USA). Graphs were drawn with Prism, version 8.4.3 (GraphPad, San Diego, USA).

Continuous variables were compared using student’s T-Test, Fisher`s exact test was used for contingency table analyses. Significance level was set at p < 0.05. The study confirms with the 1975 declaration of Helsinki and it was approved by the ethics committee of the Albert Ludwig University of Freiburg (file number 234-20).

## Results

### Patient selection and characteristics

**Fig. 1:**
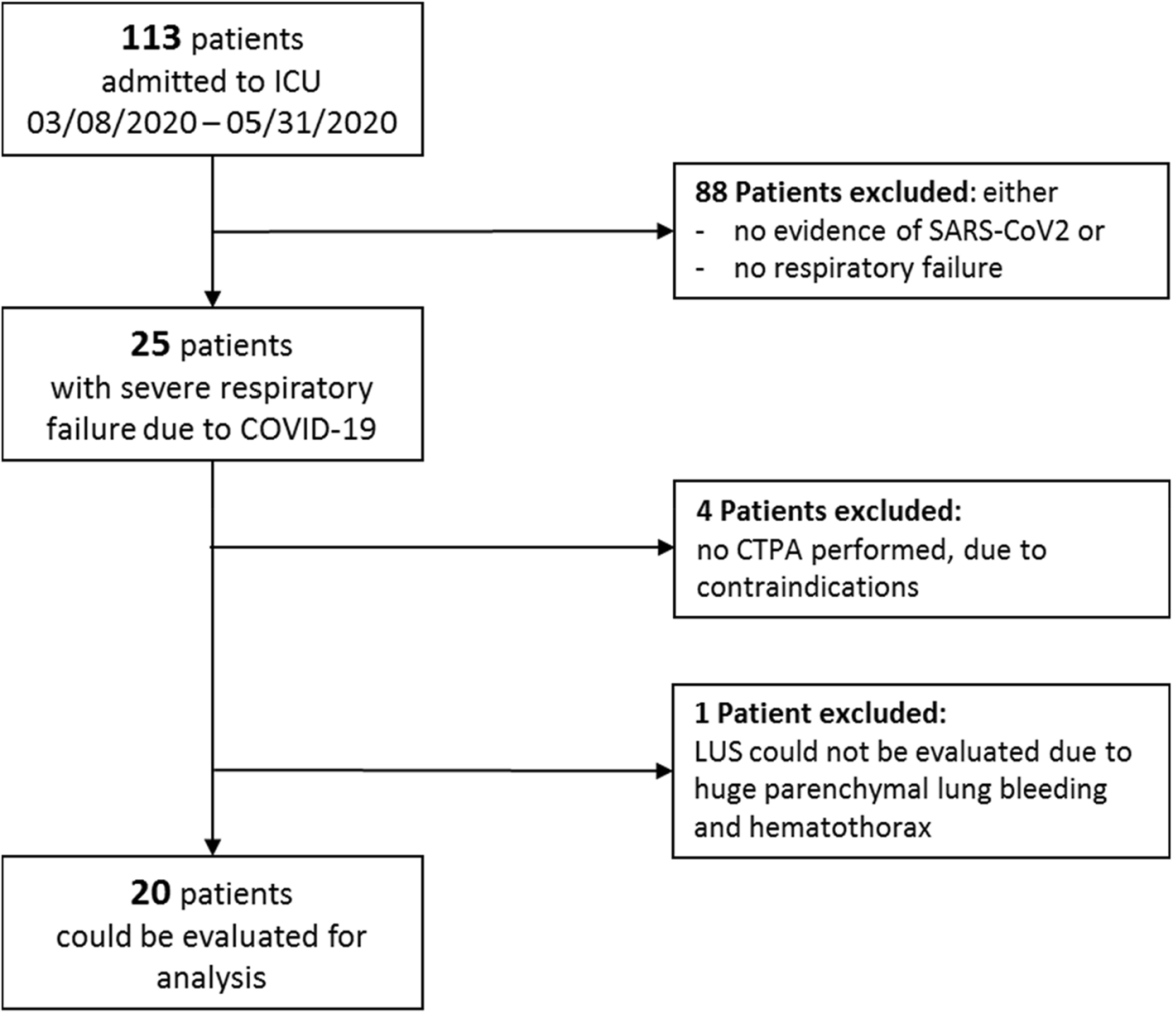
Flowchart patient selection. *CTPA* computed tomography pulmonary angiography, *COVID-19* Corona-Virus Disease 2019, *LUS* lung ultrasound

A total of 113 patients were admitted to the ICU within the observed time period, of these 25 patients were diagnosed with severe respiratory failure due to COVID-19, all of which underwent LUS. Four Patients had to be excluded from the study because they did not receive a CTPA, in one patient LUS could not be reliably evaluated due to huge parenchymal pulmonary bleeding and hemothorax (patients characteristics of patients excluded are given in supplemental material Table 1). Therefore, 20 patients could be enrolled in this study (see Fig. 1). Mean age (± S.D.) of the patients was 61.6 ± 9.9 years, SAPS II score was 48.4 ± 12.4 on day 1 after admission to ICU. Eighteen of twenty patients (90%) were intubated. Eleven from 20 (55%) patients were on ECMO during their ICU stay. For more detailed patient characteristics see Table 1.

**Table 1:** Patients characteristics of all patients, with ARDS due to COVID-19, which underwent LUS, Wells score and CTPA: the number within the two groups (PE vs. non-PE) as well as the percentage in relation to the entire group or the standard deviation are given

Characteristics	With PE	No PE	p value < 0.05*
Number	12.0 (60%)	8.0 (40%)	0.690
Age	59.0 ± 8.0	65.5 ± 11.8	0.190
Female	4.0 (20.0%)	2.0 (10.0%)	0.690
BMI (kg/m^2^)	26.9 ± 3.6	30.4 ± 8.8	0.285
ICU-mortality	5.0 (25%)	4.0 (20%)	0.713
ICU-stay (in days)	27.3 ± 26.8	30.0 ± 23.3	0.819
TISS 10—score	17.0 ± 6.7	15.4 ± 5.8	0.500
SAPS 2—score	50.0 ± 10.4	45.9 ± 15.4	0.395
d-dimers (mg/l) (at time of LUS)	16.3 ± 13.3	13.5 ± 12.6	0.579
d-dimers (mg/l) (at time of admission)	6.7 ± 6.0	4.2 ± 3.0	0.526
Wells score (at time of LUS)	2.7 ± 0.8	1.7 ± 0.5	**0.042**
Therapeutic anticoagulation (at time of admission)	1.0 (5.0%)	4.0 (20.0%)	**0.035**
Echocardiography: PAP sys (mmHg)	46.8 ± 18.9	42.6 ± 16.0	0.563
Invasive mechanical respiratory support (in days)	28.8 ± 29.4	29.1 ± 25.0	0.988
On ECMO support	7 (35%)	4 (20%)	0.713
Pre-existing co-morbidities			
Lung disorder	1.0 (5.0%)	4.0 (20.0%)	**0.035**
Tobacco smoke	3.0 (15.0%)	5.0 (25.0%)	0.094
Diabetes mellitus	3.0 (15.0%)	0.0 (0.0%)	0.125
Arterial hypertension	3.0 (15.0%)	4.0 (20.0%)	0.251
Heart failure	2.0 (10.0%)	3.0 (15.0%)	0.292
Kidney failure	1.0 (0.5%)	1.0 (5.0%)	0.761
Liver failure	0.0 (0.0%)	1.0 (5.0%)	0.210
Coagulopathy	1.0 (5.0%)	0.0 (0.0%)	0.402
Immunodeficiency	3.0 (15.0%)	0.0 (0.0%)	0.125
Obesity (BMI > 30)	2.0 (10.0%)	2.0 (10.0%)	0.648

Significant values are given in bold (p < 0.05)

### Computed tomography pulmonary angiography (CTPA)

The final diagnosis of PE was confirmed by CTPA. In total 300 lung segments were evaluated. In 12 out of 20 patients (60%), segmental and subsegmental PE were detected. In patients with PE 62 segments in total with an average of 2.90 ± 3.38 lung segments were affected (range 1 to 9). The analysis of distribution of PE on lung lobe level, showed the right lower lobe to be affected in 10 out of 12 patients with PE, followed by the right upper lobe which was affected in 8 out of 12 patients. The complete PE distribution of the entire lung is shown in Fig. 1 of the supplement.

### LUS in COVID-19

**Fig. 2:**
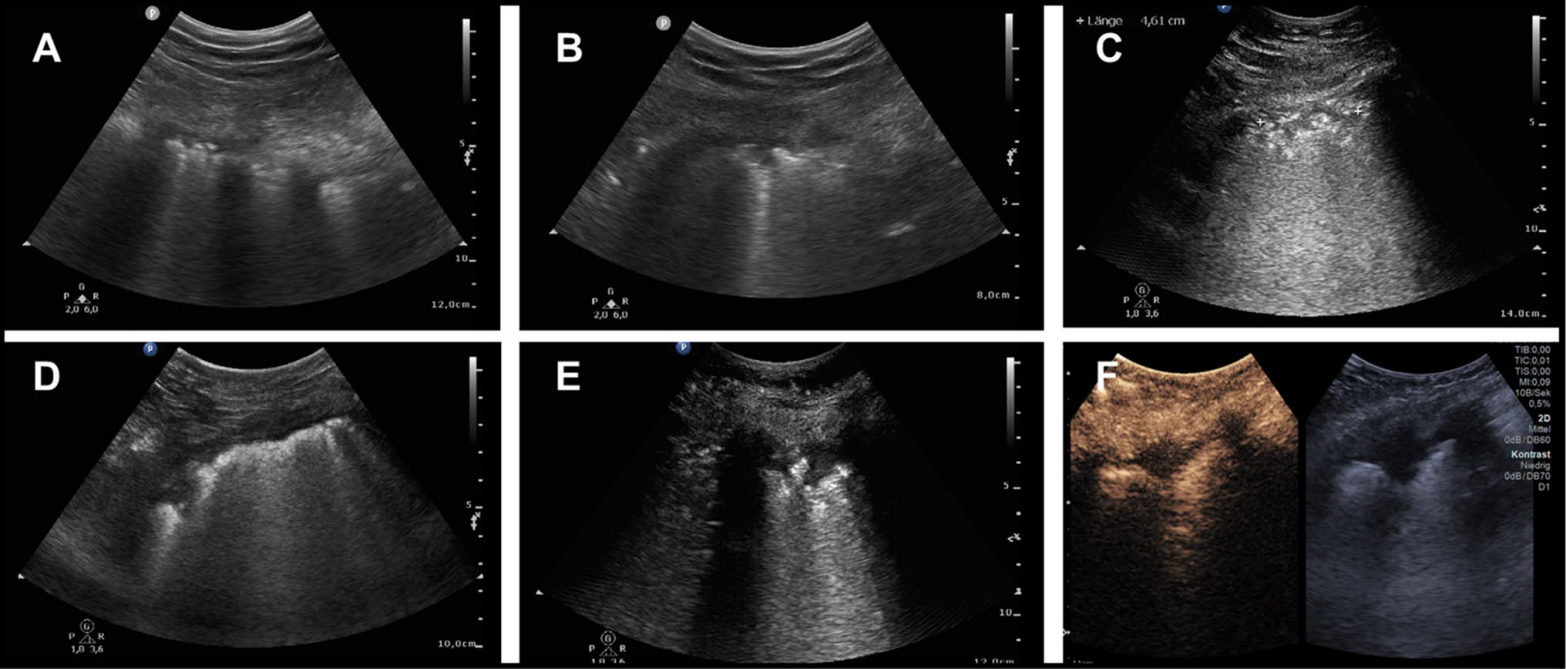
Sonographic image-examples of the pleural and subpleural changes in COVID-19 patients. **a** Typical thickening and irregularity of the pleura. **b** Small triangular subpleural consolidation < 1 cm. **c** Non-typical polygonal subpleural consolidation. **d**, **e** PE-typical triangular subpleural consolidation > 1 cm. **f** PE-typical triangular subpleural consolid.  > 1 cm, with additional documentation after contrast agent

LUS examinations showed abnormal lung ultrasound findings, with pleural abnormalities including thickening of the pleural line with pleural line irregularity in nineteen of twenty cases (95%). B-lines, in a variety of patterns including focal, multifocal, and confluent could also be documented in 19 from 20 (95%) patients. Multifocal B-lines were found in 14 patients and confluent in 11 patients, however B-line pattern was heterogeneous in individual patients. Subpleural consolidations were found in 18/20 (90%) patients. Typical consolidations with a size/depth of > 1 cm were detectable in 13/20 (65%) patients. Ten (50%) patients showed atypical consolidations (< 1 cm). Typical and atypical consolidations could occur co-dominantly in the same individual. The COVID-19-typical lung sonographic findings are summarized in Table 2. Sonographic image-examples of these different pleural morphologies are shown in Fig. 2.

**Table 2:** COVID-typical Lung ultrasound findings of the 20 patients included

Pleural line abnormalities	95% (19)
B-lines	95% (19)
Multifocal B-lines	70% (14)
Confluent B-lines	55% (11)
Supleural consolidations	90% (18)
Typical (≥ 1 cm)	65% (13)
Atypical(< 1 cm)	50% (10)
Pleural effusion	10% (2)
Pericardial effusion	5% (1)

Data are presented as percentage (number of cases with findings)

### Wells score

Average Wells score in the entire cohort was 2.3 ± 0.8. In the group with evidence of PE, the wells score was significantly higher than in the group without PE (2.7 ± 0.8 in patients with respectively 1.7 ± 0.5 in patients without PE, p = 0.042).

### PE prediction

**Fig. 3:**
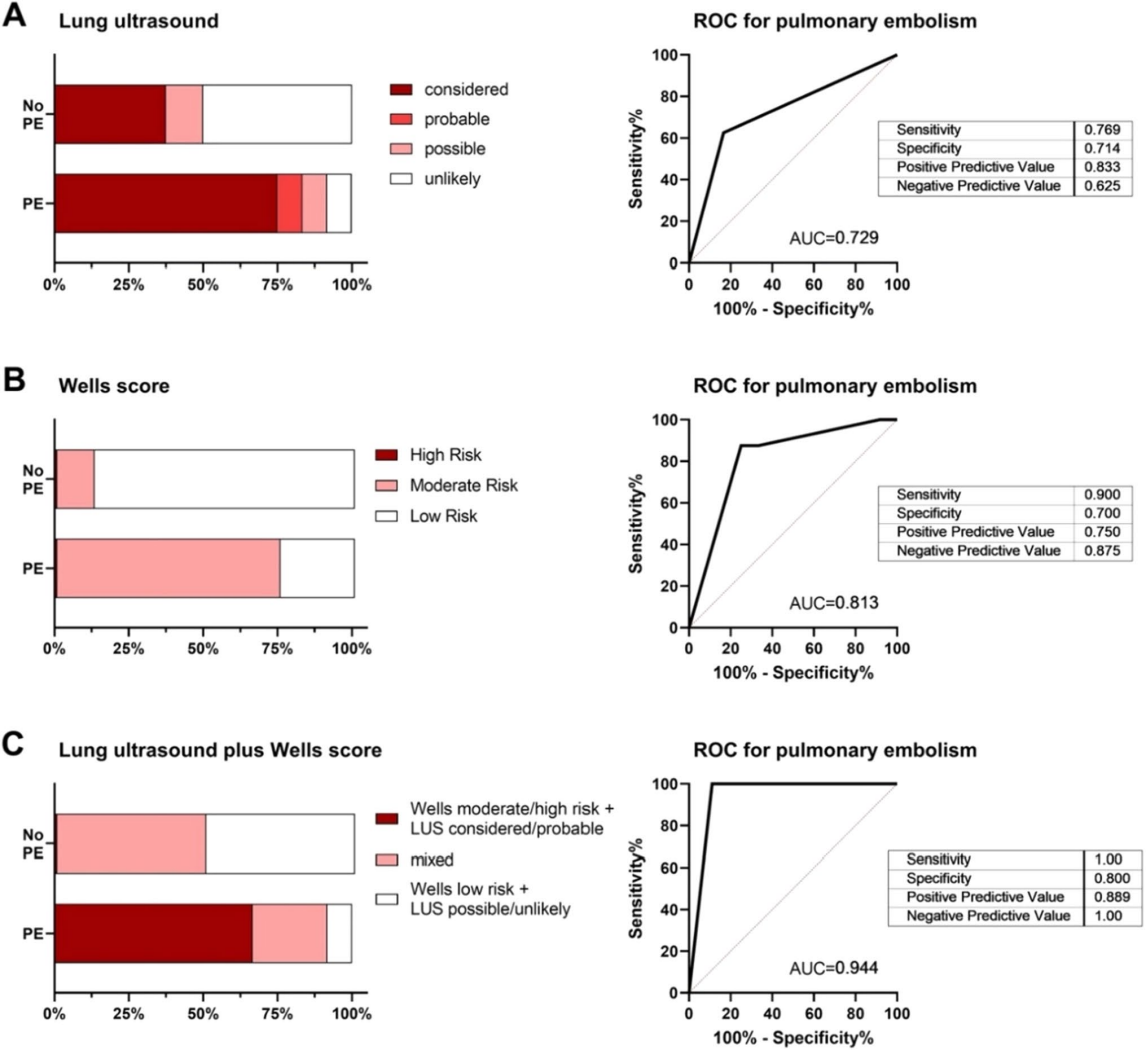
Prediction of pulmonary artery embolism by lung ultrasound and wells score

Using LUS and the criteria described above and suggested by [14], PE was considered in 12/20 (60%) patients, probable in 1/20 (5%), possible in 2/20 (10%), and unlikely in 5/20 (25%) patients. When forming a two-tier scale of probable PE (considered/probable PE in LUS versus possible/unlikely PE in LUS), pulmonary embolism could be predicted with an AUC of 0.729 and a sensitivity of 77% and a specificity of 71%, see Fig. 2. The Wells score estimated the risk for PE as very high (Wells score > 6) in 0/20 patients, as moderate (score 2–6) in 10/20 patients and as low (score < 2) in 10/20 patients. With the Wells score, PE could be predicted with an AUC of 0.813 and a sensitivity of 90% and a specificity of 70%, see Fig. 2. When combining the two modalities, comparing patients with considered/probable PE using LUS plus a Wells score ≥ 2 to patients with possible/unlikely PE using LUS plus a Wells score < 2, PE could be predicted with an AUC of 0.944 and a sensitivity of 100% and a specificity of 80%, see Fig. 3.

## Discussion

Using a standardized LUS exam, focusing on subpleural consolidations ≥ 1 cm, predicted PE in patients with COVID-19 ARDS with a specificity of 83.3% and a sensitivity of 77%.

Subpleural consolidations have been described as typical signs for COVID-19. In addition, these consolidations have been described as sonographic criteria for peripheral or segmental PE with high sensitivity and specificity (76.9% and 91.3%, respectively) [14] in non-COVID-19 patients. So far, however, LUS for detection of PE has not been validated in COVID-19. Our results might confirm the findings that were generally obtained for peripheral PE in patients without COVID-19 [14, 19, 20]. For the sonographic assessment of the PE-probability, we followed the classification suggested elegantly by Mathis et al. whoever using a two-tier scale as described above.

Lung sonography has developed considerably in recent years and is therefore widely used with regard to its clinical application. Although the method is dependent on the examiner and requires a certain amount of practice, it is easy to learn, available everywhere, can be carried out without radiation exposure or the use of contrast agent and is cost-effective compared to methods such as CT-examinations or ventilation perfusion scintigraphy. In addition, infectious patients do not have to be transported as ultrasound can be performed on the bed side (point-of-care). Since LUS can identify changes in superficial lung tissue that correlate with histopathological findings which could only be identified in CT-scan, but remain hidden in a large percentage of chest X-rays, the role of LUS in the context of viral pneumonia may be relevant [17]. In view of the recent outbreak of pneumonia from Wuhan, China, in December 2019 by the SARS-CoV2 pathogen, which is referred to as COVID-19, lung sonography proved to be useful [7, 17, 18]. Typical signs for COVID-19 have been defined, which might be particularly helpful in the emergency rooms for diagnosis and triage [17, 18]. Our study confirms the signs described by Peng et al. in all but one COVID-19 patient included in the study thou consolidations are not exclusive for COVID-19.

However, with the LUS alone we might overestimate the occurrence of PE (false-positive 3/8 patients, 37.5%). When increasing the threshold to 2 subpleural consolidations ≥ 1 cm for diagnosis for PE, sensitivity drops. In order to improve specificity, a combination with a second method could be useful. The Wells score has also not yet been validated in patients with COVID-19. Although the results of the Wells score were very homogeneous in our cohort, we could predict PE with the Wells score alone with an AUC of 0.813, a sensitivity of 90% and a specificity of 70%. When combining the two modalities, comparing patients with considered/probable PE using LUS plus a Wells score ≥ 2 to patients with possible/unlikely PE using LUS plus a Wells score < 2, pulmonary embolism could be predicted with an AUC of 0.944 and a sensitivity of 100% and a specificity of 80%.

The authors believe that data available strongly suggests that every intensivist should be able to perform an ultrasound investigation of the chest. At the bedside, it is reasonable that all patients with COVID-19 ARDS should undergo repeated LUS during the ICU course, not only for detection of PE. In patients with two or more subpleural consolidation, a CTPA should be discussed since PE is very likely. In patients with contraindication to CTPA and a Wells-Score > 2.5, our data suggests PE is most likely and anticoagulation should be considered.

## Limitations

We acknowledge the preliminary nature of these findings, including its retrospective nature and limited sample size. As we included only patients with COVID-19 ARDS undergoing CTPA in this research (neglecting patients without CTPA and those without evaluable LUS), findings presented here cannot necessarily be extrapolated and have to be validated in a larger, and more heterogeneous COVID-19 cohort as well as a non-COVID cohort. Because pleural defects are nonspecific in B‐mode, other examiners used contrast agent ultrasound (CEUS), with which the subpleural consolidations could be depicted even better. Trenker et al. found that, despite the lack of definite confirmation of PE on CT, peripheral subpleural consolidations with no or inhomogeneous enhancement on CEUS to be highly suggestive of embolic consolidations [21]. A follow-up study by the same team used histological examination of six cases, and pulmonary infarction was found in all of them [22]. This case series underlines that peripheral PEs are not necessarily discovered in the CTPA. When considering the recently published overview by Bérangère et al. [23] of the pathophysiological relationships of hemostasis in critically ill COVID-19 patients and the high risk of developing macro- and microthrombi due to hypercoagulability and endotheliopathy, it seems quite possible that there is not an overestimation of the PE-frequency in LUS, but instead an underestimation using CTPA as possible reasons for the false positive cases in the LUS. Unfortunately, contrast agent ultrasound exams were not part of our standardized LUS examination.

## Conclusion

Large subpleural consolidations ≥ 1 cm detected in lung ultrasound were found frequently in COVID-19 ARDS patients with pulmonary embolism. In combination with a Wells score > 2, PE could be predicted in COVID-19 ARDS with a sensitivity of 100% and a specificity of 80%. This data might advocate a combination of LUS and the Wells score as screening tool for PE in COVID-19 ARDS.

## Electronic supplementary material

Below is the link to the electronic supplementary material.

Electronic supplementary material 1 (DOCX 18 kb)

## Data Availability

The datasets of this study are available from the corresponding author on reasonable request.

## Abbreviations

PE: Pulmonary embolism
ARDS: Acute respiratory distress syndrom
LUS: Lung ultrasound
CTPA: Computed tomography pulmonary angiography
ECMO: Veno-venous extracorporeal membrane oxygenation
COVID-19: Corona-Virus Disease 2019
SD: Standard deviation
BMI: Body mass index
PAP sys: Systolic pulmonary arterial pressure
SAPS2: Simplified acute physiology core2
TISS: Therapeutic Intervention scoring system
ICU: Intensive care unit
DVT: Deep vein thrombosis
LMWH: Low molecular weight heparin
ROC: Receiver operating characteristic-curve
