# Supplementary material for: Combining lung ultrasound and Wells score for diagnosing pulmonary embolism in critically ill COVID-19 patients
Source: J Thromb Thrombolysis. 2020 Nov 3;52(1):76–84. doi: 10.1007/s11239-020-02323-0 (PMC7608377; doi:10.1007/s11239-020-02323-0)
Supplement: Supplementary file 1 — Electronic supplementary material 1 (DOCX 18 kb) [file 11239_2020_2323_MOESM1_ESM.docx]

| **Characteristics** | Included in study | Excluded from study | p value <0.05* |
| --- | --- | --- | --- |
| number | 20.0 (100%) | 5.0 (100%) |  |
| age | 61.6 ± 10.0 | 58.2 ± 11.7 | 0.5163 |
| female | 6.0 (30.0%) | 1.0 (20.0%) | 1.0000 |
| BMI [kg/m²] | 28.3 ± 6.2 | 32.3 ± 5.0 | 0.1961 |
| ICU-mortality | 9.0 (45%) | 2.0 (40%) | 1.0000 |
| ICU-stay [in days] | 28.4 ± 24.8 | 25.6 ± 26.9 | 0.8260 |
| TISS 10 - Score | 16.4 ± 6.2 | 15.0 ± 10.8 | 0.7015 |
| SAPS 2 - Score | 48.4 ± 12.4 | 42.0 ± 15.4 | 0.3123 |
| d-dimers [mg/l] (at time of LUS) | 15.2 ± 12.8 | 10.2 ± 8.5 | 0.4194 |
| d-dimers [mg/l] (at time of admission) | 5.7 ± 5.1 | 2.6 ± 2.6 | 0.2057 |
| wells score (at time of LUS) | 2.3 ± 0.8 | 2.0 ± 1.1 | 0.4922 |
| therapeutic anticoagulation (at time of admission) | 4.0 (20.0%) | 1.0 (20.0%) | 1.0000 |
| Echocardiography: PAP sys. [mmHg] | 42.6 ± 16.0 | 47.5 ± 3.5 | 0.5092 |
| invasive mechanical respiratory support [in days] | 28.9 ± 27.0 | 37.0 ± 36.6 | 0.5805 |
| on ECMO support | 11 (55%) | 2 (40%) | 0.6447 |
| pre-existing co-morbidities: |  |  |  |
| Lung disorder | 5.0 (25.0%) | 0.0 (0.0%) | 0.5440 |
| Tobacco smoke | 8.0 (40.0%) | 1.0 (20.0%) | 0.6206 |
| Diabetes mellitus | 3.0 (15.0%) | 3.0 (60.0%) | 0.0698 |
| Arterial hypertension | 7.0 (35.0%) | 3.0 (60.0%) | 0.3577 |
| Heart failure | 5.0 (25.0%) | 1.0 (20.0%) | 1.0000 |
| Kidney failure | 2.0 (10.0%) | 1.0 (20.0%) | 0.5043 |
| Liver failure | 1.0 (5.0%) | 0.0 (0.0%) | 1.0000 |
| Coagulopathy | 1.0 (5.0%) | 0.0 (0.0%) | 1.0000 |
| Immunodeficiency | 3.0 (15.0%) | 0.0 (0.0%) | 1.0000 |
| Obesity (BMI >30) | 4.0 (20.0%) | 4.0 (80.0%) | **0.0235** |

**Table 3:**  Patients characteristics of all patients, with ARDS due to COVID-19.

Column 1: all patients with COVID19-ARDS who could be included in the study.

Column 2: patients who were excluded due to non-evaluable LUS or the lack of CTPA
